# Targeting MC1R depalmitoylation to prevent melanomagenesis in redheads

**DOI:** 10.1038/s41467-019-08691-3

**Published:** 2019-02-20

**Authors:** Shuyang Chen, Changpeng Han, Xiao Miao, Xin Li, Chengqian Yin, Junrong Zou, Min Liu, Shanshan Li, Lukasz Stawski, Bo Zhu, Qiong Shi, Zhi-Xiang Xu, Chunying Li, Colin R. Goding, Jun Zhou, Rutao Cui

**Affiliations:** 1 Shandong Provincial Key Laboratory of Animal Resistance Biology, Institute of Biomedical Sciences, College of Life Sciences, Shandong Normal University, 250014 Jinan, Shandong China; 2 Department of Pharmacology and Experimental Therapeutics, Boston University School of Medicine, Boston, MA 02118 USA; 3 Yueyang Hospital, Shanghai University of Traditional Chinese Medicine, 201203 Shanghai, China; 4 Innovation Research Institute of Traditional Chinese Medicine, Shanghai University of Traditional Chinese Medicine, 201203 Shanghai, China; 5 Arthritis Center/Section of Rheumatology, Boston University School of Medicine, Boston, MA 02118 USA; 6 Department of Dermatology, Xijing Hospital, The Fourth Military Medical University, 710000 Xi’an, Shaanxi China; 7 Division of Hematology and Oncology, Department of Medicine, Comprehensive Care Center, University of Alabama at Birmingham, Birmingham, AL 35294 USA; 8 Ludwig Institute for Cancer Research, University of Oxford, Headington, Oxford, OX3 7DQ UK

## Abstract

Some genetic melanocortin-1 receptor (MC1R) variants responsible for human red hair color (RHC-variants) are consequently associated with increased melanoma risk. Although MC1R signaling is critically dependent on its palmitoylation primarily mediated by the ZDHHC13 protein-acyl transferase, whether increasing MC1R palmitoylation represents a viable therapeutic target to limit melanomagenesis in redheads is unknown. Here we identify a specific and efficient in vivo strategy to induce MC1R palmitoylation for therapeutic benefit. We validate the importance of ZDHHC13 to MC1R signaling in vivo by targeted expression of ZDHHC13 in C57BL/6J-MC1R^RHC^ mice and subsequently inhibit melanomagenesis. By identifying APT2 as the MC1R depalmitoylation enzyme, we are able to demonstrate that administration of the selective APT2 inhibitor ML349 treatment efficiently increases MC1R signaling and represses UVB-induced melanomagenesis in vitro and in vivo. Targeting APT2, therefore, represents a preventive/therapeutic strategy to reduce melanoma risk, especially in individuals with red hair.

## Abstract (web summary)

Melanocortin-1 receptor is a palmitoylated protein and variants of the receptor are associated with red hair colour and susceptibility to melanoma. Here, the authors describe a method to enhance the palmitoylation of the receptor, which can inhibit melanomagenesis in mice.

## Introduction

Although melanoma accounts only for 1% of skin cancer, it causes the majority of skin cancer-associated deaths. Caucasians in the United States have an approximately 25-fold higher risk of developing melanoma than African Americans, and melanoma risk is almost tripled again in redheads compared to other Caucasians^1^. The melanocortin-1 receptor (MC1R), a well-known G protein-coupled receptor (GPCR), is the key regulator of hair and skin pigmentation. Upon ultraviolet (UV) irradiation, MC1R is bound by keratinocyte-derived α-melanocyte-stimulating hormone (α-MSH) to activate cAMP signaling, enhance melanin production in melanocytes, and stimulate DNA-damage repair. Human studies and mouse models have demonstrated that MC1R genetic variants are tightly correlated with phenotypes, such as red hair, fair skin, freckling, UV irradiation sensitivity, and melanoma risk. These variants are defined as red-hair-color (RHC) variants^2,3^. R151C, R160W, and D294H are three most common “strong” red hair variants as they make up >60% of all red hair cases^4–7^. R151C and R160W are reported to be associated with red hair, fair skin, and freckles, while D294H only associates with the red hair and freckles phenotype in Caucasians^4–7^. These MC1R RHC variants lead to pheomelanin production and make redheads more susceptible to skin cancer^8,9^.

While many independent studies have demonstrated that melanoma risk is higher in people who carry MC1R RHC variants, the underlying mechanisms are only just being elucidated. The increased melanoma risk attributable to MC1R RHC variants may arise in part through skin pigmentation since pheomelanin in redheads contributes to melanomagenesis through UV radiation (UVR)-independent oxidative damage^8,10^. However, some MC1R variants are not linked with a red-hair phenotype but remain associated with elevated risk of developing melanoma^11–13^. In Caucasians with melanoma, MC1R variants were detected in 15–33% of dark-haired subjects and 42% of dark-eyed subjects; MC1R variants possibly negate the protective effects of dark pigment. Beyond pigmentation, MC1R plays additional roles in melanoma development. For example, MC1R controls ultraviolet B (UVB)-induced G1-like cell cycle arrest and subsequent onset of premature senescence in melanocytes, abrogation of which contributes to melanoma development^14^. Moreover, MC1R signaling plays an important role in promoting efficient DNA-damage repair^10,15–20^. Collectively, these observations raise a key question: can therapeutic intervention directed toward enhancing MC1R signaling reverse the increased melanoma risk associated with MC1R RHC variants?

One attractive approach is to increase MC1R palmitoylation, a modification common in GPCRs in which reversible addition of palmitic acid to a cysteine residue of the C-terminal tail or the intracellular loops profoundly affects their structure, stability, membrane localization, or interaction with partner proteins. MC1R palmitoylation is mediated by ZDHHC13 and is essential for activating MC1R signaling^9^. However, the enzyme(s) required for MC1R depalmitoylation have yet to be identified, though palmitoyl-protein thioesterases (PPTs), including acyl-protein thioesterase-1 (APT1), APT2, and other serine hydrolases^21–23^, represent potential candidates. Importantly, MC1R RHC variants exhibit reduced palmitoylation and consequently defective signaling^9^. As such, inhibiting MC1R depalmitoylation should enhance signaling from this GPCR and prevent the increased melanoma risk associated with MC1R RHC variants.

Here we report that ZDHHC13 expression correlates with MC1R signaling and survival in human melanoma and that its expression can rescue MC1R RHC variant signaling in vitro and in vivo to suppress UVR-induced melanomagenesis. Significantly, we reveal that MC1R de-palmitoylation is catalyzed by APT2 and consequently ML349, an APT2 inhibitor, rescues defects in MC1R RHC variant signaling and offers a potential avenue to an effective melanoma prevention strategy.

## Results

### Clinical relevance of ZDHHC13 and MC1R signaling in human melanomas

**Fig. 1:**
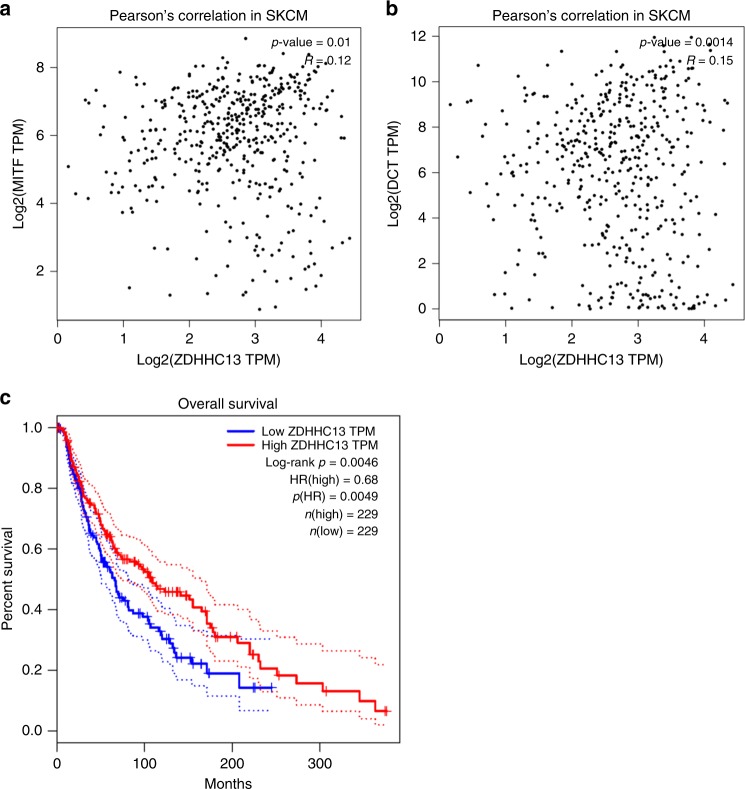
Clinical relevance of ZDHHC13 and melanocortin-1 receptor signaling in human melanomas. **a**, **b** Pearson’s correlation between ZDHHC13 and MITF (**a**) or DCT (**b**) in The Cancer Genome Atlas (TCGA) SKCM. Plots show the Spearman’s correlation and MITF (left) or DCT (right) with ZDHHC13 mRNA level from RNA-seq data in TCGA melanoma calculated by GEPIA (Gene Expression Profiling Interactive Analysis). *R* and *p* values are shown. **c** Melanoma patient survival analysis based on ZDHHC13 expression (cutoff = 50%). All patients in the TCGA melanoma study were divided according to the expression level of ZDHHC13 (higher or lower level than median expression value of all patients)

Although the protein-acyl transferase ZDHHC13 catalyzes MC1R palmitoylation to activate MC1R signaling^9^, the clinical significance of this observation is not known. By using RNA sequencing (RNA-seq) data from the human The Cancer Genome Atlas (TCGA) melanoma cohort and GEPIA (Gene Expression Profiling Interactive Analysis)^24^, we found that the mRNA levels of ZDHHC13 positively correlated with those of well-known targets downstream from MC1R signaling (Fig. 1a, b), including MITF and DCT^25^. MITF is the master regulator of melanocyte development and regulates multiple cellular processes, including promoting survival, differentiation and proliferation, and suppressing senescence and melanoma invasion^26–30^. In addition, we used TCGA clinical data and GEPIA^24^ and found that high mRNA levels of ZDHHC13 are associated with a survival benefit in melanoma patients (Fig. 1c). These results suggest that higher expression levels of ZDHHC13 is correlated with stronger activation of MC1R signaling in human melanomas.

### Transgenic ZDHHC13 increases MC1R^R151C^ signaling in vivo

**Fig. 2:**
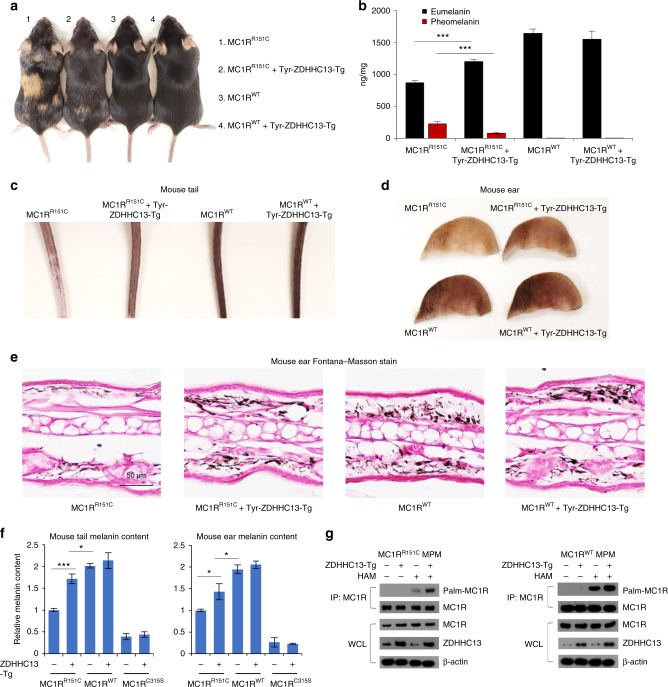
Transgenic ZDHHC13 increases MC1R^R151C^ signaling in vivo. **a** Images of mice with the indicated genotypes. All mice are on the C57/BL6J background. **b** Measurement of eumelanin and pheomelanin content in the whole back skin collected from the indicated mice shown in **a**. Eumelanin and pheomelanin were quantified by high-performance liquid chromatography based on the level of pyrrole-2,3,5-tricarboxylic acid (PTCA) by alkaline hydrogen peroxide oxidation of eumelanin and 4-amino-3-hydroxyphenylalanine (4-AHP) by hydriodic acid reductive hydrolysis of pheomelanin, respectively. Final results were determined by a conversion as described (eumelanin = 45 × PTCA, pheomelanin = 9 × 4-AHP). Three independent mice were used. **c** Tails and **d** ears of the indicated mice as shown in **a**. **e** Fontana–Masson staining of the indicated ear sections as shown in **d**. **f** Melanin quantification of the indicated tail and ear samples as shown in **c**, **d**. Three independent mice were used. **g** Melanocytes were isolated from the dorsolateral skin of 3.5-day postnatal mice. Total protein was extracted from mouse primary melanocytes and was then used for immunoprecipitation (IP) with specific anti-melanocortin-1 receptor antibodies. Acyl biotin exchange reaction and immunoblot analysis using the indicated antibodies were performed subsequently. *p < 0.05, ***p < 0.001, unpaired Student’s *t* test. Error bars represent ± s.d.

The role of ZDHHC13 as the key protein s-acyl transferase for MC1R was previously identified in vitro^9^. However, whether it plays a similar role in regulating MC1R and its downstream signaling in pigment production and melanomagenesis in vivo remain unclear. To address this issue, we developed a transgenic mouse with melanocyte-specific ZDHHC13 expression controlled by the mouse Tyr enhancer/promoter^31^ using the same strategy used by us previously to generate the MC1R RHC variant mice (C57BL/6J-MC1R^e/e^J/MC1R^R151C^-Tg, as R151C is the most common “strong” red hair allele in humans^4–7^) (Supplementary Fig. 1a). The ZDHHC13 expression construct was injected into single-cell embryos of C57BL/6J mice and the resulting transgenic ZDHHC13 was genotyped by PCR (Supplementary Fig. 1b). We noted that Tg-ZDHHC13 mice exhibited higher skin eumelanin/pheomelanin ratios than control mice (Fig. 2a, b). We next selected a Tg-ZDHHC13 mouse with medium levels of exogenous ZDHHC13 expression to cross with our previously generated MC1R RHC variant mice and palmitoylation-defective MC1R mutant C315S mice^9^ to generate Tg-ZDHHC13/MC1R RHC variant mice. The offspring that express the MC1R^R151C^ variant show a mosaic coat color, whereas those that express MC1R^C315S^ exhibit red hair phenotype similar to MC1R^e/e^ mice^9^. Tg-ZDHHC13/MC1R^R151C^ mice exhibited a darker coat color than Tg-MC1R^R151C^ mice (Fig. 2a**)**, with uniform pigmentation in individual hairs (Supplementary Fig. 1c). However, no obvious fur/skin color change was observed in MC1R^WT^ or transgenic expressing the non-palmitoylatable MC1R^C315S^ mutant, with or without transgenic ZDHHC13 (Fig. 2a and Supplementary Fig. 1d)^9^. These results suggest that exogenous ZDHHC13 overexpression rescues eumelanin production in MC1R RHC variant mice in vivo.

To further test the in vivo effect of ZDHHC13 on MC1R RHC variants, we collected mouse ears and tails, where epidermal melanocytes are located^32^, and evaluated eumelanin production. Enhanced pigmentation was observed in the ears and tails of the Tg-ZDHHC13/MC1R^R151C^ mice compared to MC1R^R151C^ alone (Fig. 2c, d**)**, a result confirmed by Fontana–Masson staining (Fig. 2e and Supplementary Fig. 1e) and biochemical assays (Fig. 2f). Notably, Tg-ZDHHC13 expression did not enhance eumelanin production in MC1R^WT^ and MC1R^C315S^ mice (Fig. 2c–f and Supplementary Fig. 1d, e). As the number of melanocytes in the hair follicle in Tg-MC1R-variants and Tg-ZDHHC13 was not altered compared to the non-Tg-ZDHHC13 controls (Supplementary Fig. 1f–j), the increased eumelanin production observed did not arise owing to ZDHHC13-stimulation of melanocyte proliferation.

To identify whether transgenic ZDHHC13 activated MC1R palmitoylation in vivo, we used the acyl-biotin exchange (ABE) palmitoylation assay to detect palmitoylated MC1R protein in mouse primary melanocytes (MPM) isolated from 3.5-day-old Tg-ZDHHC13/MC1R^R151C^ mice. MC1R proteins extracted from the isolated MPMs were pulled down with specific anti-MC1R antibodies and then treated sequentially with *N*-ethylmaliemide and hydroxylamine to expose palmitoylated cysteines. Streptavidin was used to detect biotin-BMCC-labeled MC1R palmitoylation protein. We found that Tg-ZDHHC13 slightly increased MC1R protein palmitoylation in MC1R^WT^ but remarkably enhanced MC1R protein in Tg-MC1R^R151C^ mice (Fig. 2g). These results suggest that ZDHHC13 overexpression physiologically rescues MC1R RHC-induced “red hair” phenotype by increasing MC1R palmitoylation in a mouse model.

### Inhibition of UVB-induced melanomagenesis in redheads in vivo

**Fig. 3:**
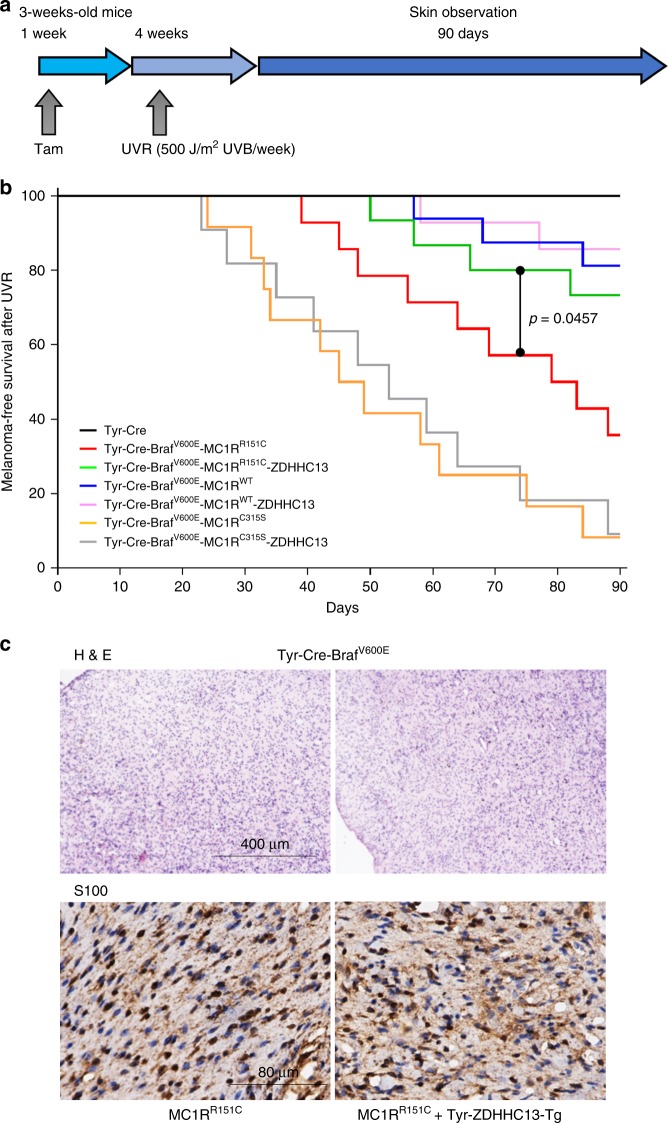
Transgenic ZDHHC13 inhibits ultraviolet B (UVB)-induced melanomagenesis in redheads in vivo. **a** Schematic for UVB-induced melanoma development in mice. **b** Kaplan–Meier plot showing melanoma-free survival of the indicated mice. Ninety days after the final UV radiation, melanoma was diagnosed in 92% (11/12), 64% (9/14), and 19% (3/16) of mice with MC1R^C315S^, MC1R^R151C^, and MC1R^WT^. Melanoma incidence was much lower in the Tg-ZDHHC13/MC1R^R151C^ mice than in the MC1R^R151C^ mice (27% (4/15) vs. 64% (9/14), log-rank test, *p* = 0.0457). **c** Hematoxylin and eosin staining of histological sections and immunohistochemistry staining of S100 of representative cutaneous melanomas. Genotypes were as indicated

To investigate the potential role for ZDHHC13 in tumor formation, Tyr-Cre-BRAF^CA^ mice (B6.Cg-Braf^tm1Mmcm^ Tg(Tyr-cre/ERT2)Bos/BosJ)^33^ were crossed with the Tg-ZDHHC13/MC1R^R151C^ mice to get Tyr-Cre-BRAF^CA^/Tg-ZDHHC13/MC1R^R151C^ mice. Tyr-Cre-ERT2 fusion gene^31^ activity was then induced by tamoxifen administration to activate BRAF^V600E^ expression specifically in melanocytes. Mice were then given 500 J/m^2^ UVB irradiation, the minimal erythema dose (MED) of UVB in mice^9,34,35^, once each week for 4 weeks and then observed for melanoma incidence for a further 90 days (Fig. 3a). Melanomas were observed in the UVB-exposed Tyr-Cre-BRAF^CA^/Tg-ZDHHC13/MC1R^R151C^ mice, with the Tyr-Cre-BRAF^CA^/Tg-ZDHHC13 mice and Tyr-Cre-BRAF^CA^/Tg-MC1R^R151C^ mice serving as controls. Melanoma was first diagnosed 24, 39, and 57 days after the final UV irradiation in mice expressing the palmitoylation defective MC1R (MC1R^C315S^), MC1R RHC variant (MC1R^R151C^), and MC1R^WT^ respectively, indicating that melanoma incidence was associated with MC1R status. Ninety days after the final UVR treatment, melanoma was diagnosed in 92% (11/12), 64% (9/14), and 19% (3/16) of mice with MC1R^C315S^, MC1R^R151C^, and MC1R^WT^, respectively (Fig. 3b). More importantly, our data indicated that melanoma incidence was much lower and with delayed first diagnosis (day 50 compared to day 39) in the Tg-ZDHHC13/MC1R^R151C^ mice than in the MC1R^R151C^ mice (27% (4/15) vs. 64% (9/14), log-rank test, *p* = 0.0457; Fig. 3b). All melanomas diagnosed in these variant mice displayed similar morphological and histologic features (Fig. 3c and Supplementary Fig. 2a). Previous report showed that UVR accelerates BRAF^V600E^-induced melanomagenesis by inducing UVR-signature C>T mutations at mouse Trp53, including H39Y, S124F (homolog to human TP53 S127), R245C (homolog to human TP53 R248), and R270C (homolog to human TP53 R273)^36^. S127, R248, and R273 are all human melanoma TP53 mutation hotspots. To test the mutations in our model, we sequenced PCR fragments covering these Trp53 mutation spots. Mutations in Trp53 (S124F, R245C, or R270C) were detected in 4/4 Tyr-Cre-Braf^V600E^-MC1R^R151C^ melanomas but only 1/4 (R270C) in Tyr-Cre-Braf^V600E^-MC1R^R151C^-ZDHHC13 mice melanomas (Supplementary Fig. 2b). Our results therefore show that ZDHHC13-activated MC1R palmitoylation plays a critical role in melanoma prevention in vivo.

### Inhibition of APT2 inhibits UVB-induced melanomagenesis in vivo

**Fig. 4:**
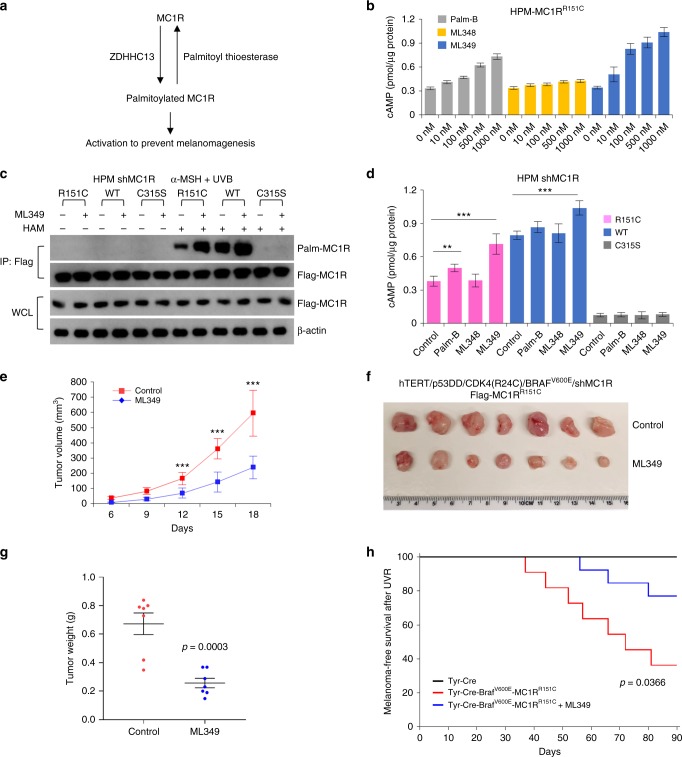
Targeted inhibition of APT2 inhibits ultraviolet B (UVB)-induced melanomagenesis in vivo. **a** Illustration of the dynamic melanocortin-1 receptor (MC1R) palmitoylation/depalmitoylation cycle. **b** cAMP levels in human primary melanocytes (HPMs) after treatment with increasing concentrations of the indicated inhibitors. HPMs in which endogenous MC1R is stably depleted using shMC1R were infected with Flag-MC1R^R151C^ and then treated with 1 μM α-melanocyte-stimulating hormone (α-MSH) and indicated inhibitors for 3.5 h. The resulting cells were harvested for a cAMP immunoassay. The data were compiled from five independent experiments. **c** MC1R-depleted HPMs were infected with the indicated Flag-MC1R-encoding retroviruses and then pretreated with 1 μM α-MSH and 100 nM ML349 for 30 min followed by 100 J/m^2^ UVB irradiation. The resulted cells were harvested for immunoprecipitation, acyl-biotin exchange, and immunoblotting analysis with the specific antibodies as indicated 3 h after UVB exposure. **d** cAMP levels in MC1R-depleted HPMs expressing the indicated Flag-MC1R encoding retroviral constructs and pretreated with 1 μM α-MSH and 100 nM inhibitors for 30 min followed by 100 J/m^2^ UVB irradiation. The resulted cells were harvested for cAMP immunoassay 3 h after UVB exposure. Data were compiled from five independent experiments. **e**–**g** Growth curves (**e**), dissected tumors (**f**), and tumor weight (**g**) for the xenograft experiments. MC1R-depleted hTERT/p53DD/CDK4(R24C)/BRAF^V600E^ melanocytes were infected with the indicated Flag-MC1R encoding retroviral constructs. Cells were preincubated with 1 μM α-MSH and 100 nM inhibitors for 30 min before being irradiated with 20 J/m^2^ UVB. After 24 h, 3 × 10^6^ cells were inoculated subcutaneously into each flank of nude mice. Visible tumors were measured at the indicated days. **h** Melanoma-free survival of the indicated mice. In the UVB radiation period, 5 mg/kg ML349 was injected intraperitoneally into mice prior to the treatment with UV. Ninety days after the final UVR, melanoma was diagnosed in 23% (3/13) or 64% (7/11) of mice with or without ML349 treatment, respectively (*p* = 0.0366). **p < 0.01, ***p < 0.001, unpaired Student’s *t* test. Error bars represent ± s.d.

The results so far present a potential therapeutic anti-melanoma strategy. Palmitoylation is a dynamic and reversible process controlled both by palmitoyl acyltransferases, which mediate palmitoylation, and PPTs, which remove this modification (Fig. 4a and Supplementary Fig. 3a). Increasing MC1R palmitoylation in vivo would potentially diminish melanoma incidence, especially for redheads in whom MC1R signaling is compromised. To increase MC1R palmitoylation, we compared the effects of three commercially available depalmitoylation inhibitors, including Palmostatin B (Palm-B)^23^, ML348, and ML349^37^. Palm-B is a newly described inhibitor of the deacylating enzymes APT1, APT2, and ABHD17 and therefore specifically inhibits depalmitoylation^38^, whereas ML348 is a newly described selective APT1 inhibitor and ML349 is a selective APT2 inhibitor^37^.

We first compared the effect of increasing concentrations (10, 100, 500, 1000 nM) of Palm-B, ML348, or ML349 on cAMP levels in human primary melanocytes (HPMs) engineered to express MC1R^R151C^ together with α-MSH (1 µM). We found that ML349 was the most efficient at increasing cAMP production in melanocytes expressing the MC1R^R151C^ variant (Fig. 4b). These results suggest that ML349 potentially activates palmitoylation of MC1R and that targeted inhibition of APT2 inhibition might be a strategy to prevent melanomagenesis in redheads.

To confirm that APT2 affects MC1R palmitoylation, HPMs co-infected with HA-APT1/Flag-MC1R or HA-APT2/Flag-MC1R-expressing viruses were assessed for MC1R protein palmitoylation using the ABE assay. We found that expression of APT2, but not APT1, decreased MC1R palmitoylation and that knockdown of APT2 upregulated MC1R palmitoylation (Supplementary Fig. 3b–d). We also found using co-immunoprecipitation that MC1R specifically interacted with APT2, but not APT1 (Supplementary Fig. 3e–h). Notably, in melanocytes knockdown of APT2 stimulated palmitoylation of the MC1R^R151C^ RHC variant but not the palmitoylation-defective MC1R^C315S^ mutant (Supplementary Fig. 3i–j). Collectively, these data strongly suggest that APT2 is the key MC1R depalmitoylation enzyme.

We next asked whether APT2-regulated MC1R palmitoylation contributes to the control of UVR-induced premature senescence in melanocytes^9,14^. APT2 was ectopically expressed or knocked down in HPMs and the resulting cells irradiated with a low dose of UVB (25 J/m^2^), before assaying for senescence associated β-gal activity. We found that overexpression of APT2 augmented low-dose UVB-induced premature senescence in melanocytes engineered to express either the MC1R RHC variant or wild-type (WT) MC1R (Supplementary Fig. 4a). By contrast, knockdown of APT2 inhibited UVB-induced and MC1R RHC-augmented melanocyte premature senescence (Supplementary Fig. 4a).

Having established a key role for APT2 in regulation of MC1R RHC variant function in vivo, we next evaluated the role of the specific APT2-targeted inhibitor, ML349^37^, in activating MC1R signaling and inhibiting melanomagenesis. To this end, HPMs engineered to express stably short hairpin RNA (shRNA) targeting endogenous MC1R and re-expressing MC1R^R151C^ or WT MC1R were pretreated with ML349 (100 nM) and α-MSH (1 µM) for 30 min following 100 J/m^2^ UVB treatment. MC1R protein palmitoylation was then detected using the ABE palmitoylation assay 3 h after UV radiation. The dose of 100 J/m^2^ is the standard erythema dose (SED) of UVB, and the ambient exposure over an entire sunny summer day in Europe (Switzerland) is approximately 30–40 SED^39^. While the MC1R^C315S^ mutant was not palmitoylated as expected, ML349 inhibited depalmitoylation of both the WT MC1R and MC1R^R151C^ proteins (Fig. 4c). Stimulation of the same engineered HPMs with ML349 (100 nM) and α-MSH (1 µM) revealed that ML349 induced cAMP production in melanocytes expressing the RHC MC1R^R151C^ variant (Fig. 4d). These results confirm that ML349 is potentially an effective therapeutic agent that can rescue the defects in MC1R RHC variant cAMP signaling.

Using genetically engineered human immortal melanocytes (hTERT/p53DD/CDK4(R24C))^9,14,40^ and melanoma xenograft mouse models, we examined the potential for APT2 inhibition by ML349 to impact melanocyte malignant transformation in vitro and ex vivo. To this end, we compared the effect of the three depalmitoylation inhibitors ML349, ML348, and Palm-B on hTERT/p53DD/CDK4(R24C) melanocytes in which expression of endogenous MC1R was suppressed using shRNA and MC1R^R151C^ was reintroduced together with BRAF^V600E^. Colony-formation and soft agar assays were performed to identify malignant transformation. We found that treatment with ML349 inhibited the MC1R^R151C^ augmented BRAF^V600E^-mediated malignant transformation more efficiently than Palm-B or ML348 (Supplementary Fig. 4b, c). Similar results were obtained using soft agar assays (Supplementary Fig. 4d, e) and were further confirmed using mouse xenograft studies in which ML349 treatment markedly inhibited MC1R^R151C^-augmented BRAF^V600E^-mediated malignant transformation (Fig. 4e–g and Supplementary Fig. 4f).

The preventative effect of ML349 on melanomagenesis in redheads was validated using our previously generated MC1R RHC variant mice (Tyr-Cre-BRAF^CA^/C57BL/6J-MC1R^e/e^J/MC1R^R151C^-Tg) that were given a dose of 500 J/m^2^ UVB irradiation each week for 4 weeks and then observed for melanoma incidence for another 90 days. In the UVB radiation period, 5 mg/kg ML349 was injected intraperitoneally into mice prior to the treatment with UV. While melanoma was first diagnosed 37 days after the final UV irradiation in mice without treatment, ML349 delayed the onset of melanoma to 56 days. Furthermore, 90 days after the final UVR, melanoma was diagnosed in 23% (3/13) or 64% (7/11) of mice with or without ML349 treatment, respectively (*p* = 0.0366; Fig. 4h). In addition, mutations of Trp53 hotspots (R245C or R270C)^36^ were detected in 3/4 Tyr-Cre-Braf^V600E^-MC1R^R151C^ melanomas but not in Tyr-Cre-Braf^V600E^-MC1R^R151C^+ML349 mice melanomas (Supplementary Fig. 4g). As no strong side effects of ML349 treatment were observed, these data suggest that ML349 is a potential small molecule to inhibit melanomagenesis in redheads.

## Discussion

The activation of α-MSH/MC1R signaling in melanocytes requires palmitoylation^9^ and is engaged in three important pathways for melanoma development: pigment production^41,42^, DNA repair^15–19^, and phosphatase and tensin homolog signaling^14^. Protein palmitoylation is controlled by the balance between protein-acyl transferases and the depalmitoylating enzymes (palmitoyl protein thioesterases) that remove this modification. Although several protein-acyl transferases are expressed in melanocytes, previous work^9^ identified ZDHHC13 as the major MC1R palmitoylation enzyme. Indeed, ZDHHC13 overexpression increased the palmitoylation of MC1R variants to prevent melanocyte malignant transformation in vitro and ex vivo^9^.

There are two Tyr-CreERT2-BRAF^CA^ melanoma mouse models: the “Marais–Larue”^43^ and the “McMahon–Bosenberg”^33^ models. Both of them are conditional BRAF^V600E^ knock in mouse models, but the “Marais–Larue” homozygous BRAF^V600E^ mice are lethal during early development. Moreover, they used two different Tyr-CreERT2 mice that have different characteristics^31,44^. For instance, Tyr-CreERT2 mice published by Bosenberg and colleagues^31^ transcribe CreERT2 in melanocyte stem cells, but Tyr-CreERT2 published by Yajima and colleagues^44^ does not transcribe CreERT2 in melanocyte stem cells. Our preliminary observations were substantiated here using our newly generated transgenic ZDHHC13 mice to demonstrate that MC1R palmitoylation and downstream signaling can be activated by ZDHHC13 in vivo to suppress melanomagenesis in “McMahon–Bosenberg” model, with 500 J/m^2^ UVB irradiation per week for 4 weeks.

Although we have focused here on ZDHHC13 in melanocyte biology and MC1R signaling in particular, it seems likely that this crucial enzyme may have a broader role as a protector of skin integrity; previous studies have demonstrated that mice with a homozygous mutation in ZDHHC13, which results in a stop codon upstream of the DHHC domain, exhibit phenotypes with severe hair and skin defects^45^.

Although ZDHHC13 was identified as the primary protein-acyl transferase mediating MC1R palmitoylation, the critical MC1R depalmitoylating enzyme had not previously been identified. Two depalmitoylation enzymes, ATP1 and ATP2, are known to control protein depalmitoylation^21,46,47^. Although the two enzymes are very similar, some studies have suggested that APT1 and APT2 may not be functionally redundant and might exhibit substrate specificity. For example, APT2, not APT1, promotes GAP-43 depalmitoylation^48^, whereas APT1, but not APT2, promotes β2-adrenergic receptor depalmitoylation^49^. Similarly, it has been shown that Scribble S-palmitoylation is regulated by APT2, but not APT1^50^. In addition, the high-resolution structure study of APT2/ML349 and APT1/ML348 complex reveal different conformations in the active sites further suggested that APT1 and APT2 may have unique roles and different functions^46^. Here our biochemical studies characterized APT2 as the major depalmitoylating enzyme of MC1R in melanocytes. Our data are therefore consistent with the two enzymes possessing distinct target specificities and indicate that APT2, but not APT1, catalyzes MC1R depalmitoylation. APT1 might not act as a depalmitoylating enzyme of MC1R because it is predominantly localized in mitochondria^51^, whereas MC1R is a plasma membrane-localized GPCR. Significantly, we demonstrate that ML349, a specific APT2 inhibitor, effectively enhances MC1R palmitoylation, increases downstream signaling, and reverses the deleterious effects of the RHC MC1R^R151C^ variant.

The potential ability to modulate MC1R function for clinical benefit, especially in redheads, has long been an ambition in skin cancer biology, though to date an effective strategy has been elusive. For example, the cyclic AMP agonist forskolin can increase pigmentation and subsequently prevent melanomagenesis^25^. Unfortunately, topical administration is problematic since forskolin does not readily penetrate the epidermis providing a major barrier for its translation to clinical use. The identification here of ZDHHC13 as a critical regulator of MC1R function in vivo offers an alternative and potentially viable approach toward melanoma prevention in highly susceptible individuals. Since palmitoylation is dynamic and reversible^9,52–54^, we can envisage either increasing ZDHHC13 function or decreasing the activity of the MC1R depalmitoylation enzyme that we identify here as APT2. Since depalmitoylation inhibitors are already available, we consider inhibition of APT2 as the most promising approach.

Although the depalmitoylation inhibitor Palm-B can activate MC1R signaling^9^ and is effective against both APT1 and APT2, the two major protein-depalmitoylating enzymes^55^, it also increases palmitoylation of HRAS and NRAS^23^. While Palm-B does not affect cell viability, it does induce loss of steady-state localization of RAS proteins and phenotypic reversion of HRAS^G12V^-transformed MDCK-F3 cells^23^. However, recent activity-based protein profiling studies found that Palm-B not only inhibits APT1 and APT2 but also inactivates many other serine hydrolases, including ABHD6, ABHD16A, ABHD17A-C, PNPLA6, and FASN^38^. Thus, although Palm-B remains a popular tool to study protein palmitoylation, its limited specificity means that it is unlikely to be used clinically. On the other hand, the recent development of more selective APT inhibitors, including ML348 (selective APT1 inhibitor, Ki = 280 nM) and ML349 (selective APT2 inhibitor, Ki = 120 nM)^37^, offered a potential opportunity to increase the activity of MC1R RHC variants by increasing their palmitoylation. Our studies here indicate that targeting APT2 to rescue MC1R palmitoylation using ML349, rather than using the generic depalmitoylation inhibitor PALM-B, may represent an effective strategy for melanoma prevention, especially in redheads.

## Methods

### UVB treatment of cells

All UVB exposure was performed as previously described^9^. Briefly, cells were washed by phosphate-buffered saline (PBS) and exposed to UVB in the Stratalinker UV chamber (Stratagene, Cedar Creek, TX, USA), in which the emittance of UVB was measured by the UV photometer (UVP LLC, Upland, CA, USA). HPMs were isolated from normal discarded foreskins as previously described^9^. Isolated HPMs were cultured in Medium 254 with melanocyte supplement (Thermo Fisher Scientific Inc., Waltham, MA, USA). All cell lines used are mycoplasma negative and were authenticated.

### Animals

Mouse experiment protocols were provided by the Institutional Animal Care and Use Committee (IACUC) of Boston University School of Medicine. All mice were maintained in pathogen-free facility of Animal Science Center of Boston University Medical Campus and strictly followed the instructions by IACUC of Boston University School of Medicine. Mice were housed on a time cycle of 12 h of light and 12 h of dark and allowed free access to sterilized diet and water. The mice were monitored daily for health and distress status. For the tumorigenesis assay of engineered hTERT/p53DD/CDK4(R24C)/BRAF^V600E^ melanocytes^14,40^, 3 × 10^6^ cells were 1:1 mixed with matrigel and injected subcutaneously into the flanks of NCr nude mice (8–10 weeks, Taconic Biosciences). Tumor size was measured by caliper and did not exceed 2 cm at the largest diameter. Transgenic mice were generated as previously described^9^ by Boston University School of Medicine Transgenic and Genome Engineering Core and Mouse ES Cell & Transgenic Facility, Koch Institute for Integrative Cancer Research at Massachusetts Institute of Technology (MIT). The expression construct was designed such that the targeted gene was inserted downstream of murine tyr promoter/enhancer region. The transgene was detected by genotyping mice tail or ear DNA using the primers targeting fragment across tyr promoter/enhancer and ZDHHC13 (GGGCTATGTACAAACTCCAAGA, CAGCTTCCAAAAGCTTATCAACT). C57BL/6J-*MC1R*^*variant*^-Tg mice were generated as previous described^9^. Tyr-Cre-BRAF^CA^ mice (B6.Cg-*Braf*^*tm1Mmcm*^ Tg(Tyr-cre/ERT2)Bos/BosJ) were purchased from The Jackson Laboratory, Stock No: 017837. Mice collected for melanomagenesis assay were administered with tamoxifen (T5648) (Sigma-Aldrich, St. Louis, MO, USA) in corn oil daily by intraperitoneal injection of 0.12 mg/g for a week. The control mice received corn oil injection. Mice UVB treatment procedure was performed as described previously^9^. The mice were irradiated 1 day per week for 500 J/m^2^ UVB treatment, which is equivalent to the mouse MEDs of UVB and commonly used as a measurement for sunlight in vivo. Specifically, mice were treated with UVB in a custom-made lucite chamber (Plastic Design Corporation, MA, USA) for 12 min. Mice were allowed freedom of movement during UVB treatment. A double bank of UVB lamps were used and UVA was filtered by the chamber. The UV emittance was measured by UV photometer (UV Products, Upland, CA, USA).

### Isolation of mouse primary melanocytes

Mouse primary melanocytes were isolated from the dorsolateral skin of 3.5-day postnatal mice. The skin was washed by PBS and then digested in 0.25% trypsin overnight. The epidermal layer was separated and minced following a centrifugation at 1000 × *g* for 10 min. Lastly, the cells were cultured in cell culture incubator with 10% CO_2_ in Medium 254 with melanocyte supplement (Thermo Fisher Scientific Inc., Waltham, MA, USA) for 14 days.

### Plasmids and infection

All MC1R plasmids were generated as previously described^9,14^. To generate the expression plasmids for retroviral infection, the cDNAs were subcloned into pQCXIP (Clonetech) at the NotI/EcoRI sites, respectively. Mutants were generated by the QuickChange II Site-Directed Mutagenesis Kit (Agilent, Santa Clara, CA, USA). To knockdown MC1R or APT2 in HPMs, shRNA in pLKO.1 targeting MC1R or APT2 were co-transfected with psPAX2 and pMD2.G in HEK293 (ATCC) using Lipofectamine 3000 (Thermo Fisher Scientific Inc., Waltham, MA, USA). Lentiviruses were collected after 48 h and then used to infect cells for 24 h in the presence of polybrene (8 μg/mL) and the infected cells were selected with puromycin (2 μg/mL). To generate cells with expression of MC1R variants or APT2, HEK293 cells were co-transfected with MC1R variants or APT2 in pQCXIP, VSV-G, and pUMVC plasmids using Lipofectamine 3000 (Thermo Fisher Scientific Inc., Waltham, MA, USA). Retroviruses were collected after 48 h and cells were infected with retroviruses in the presence of polybrene (8 μg/mL). After 24 h, cells were selected with puromycin (2 μg/mL). shRNA constructs targeting human MC1R (RHS4533-EG4157) and human APT2 (RHS4533-EG11313) were purchased from Open Biosystems (GE Healthcare Dharmacon, Inc., Lafayette, CO, USA). The most efficient knockdown of shMC1R (AAATGTCTCTTTAGGAGCCTG) and shAPT2 (AAGAAATTCCTTCACAGCTGC) were used in experiment.

### Western blot and immunoassay

Western blot, immunoprecipitation, and the ABE palmitoylation assay were performed as previously described^9,14^. Lysis buffer contains 50 mM Tris pH 7.4, 1% Triton X-100, 0.5 mM EDTA, 0.5 mM EGTA, 150 mM NaCl, 10% glycerol, 1 mM phenylmethanesulfonylfluoride, and protease inhibitor cocktail (Pierce, Thermo Fisher Scientific Inc., Waltham, MA, USA). The supernatant was collected after centrifugation at 15,000 × *g* for 15 min at 4 °C, and cell lysate was precleared by 20 µL Protein G Agarose Beads (Thermo Fisher Scientific Inc., Waltham, MA, USA) and then incubated with primary antibodies overnight at 4 °C or Flag/HA beads (Sigma-Aldrich) for 2 h. Streptavidin-HRP (1:2000) (21130) were purchased from Thermo Fisher Scientific Inc. Anti-ZDHHC13 (1:1000) (ab28759), anti-MC1R (1:1000) (ab125031), and anti-APT2 antibodies (1:500) (ab151578, ab87231) were purchased from Abcam. Anti-MC1R antibody (N-19) (sc-6875) (1:500) was purchased from Santa Cruz Biotechnologies, Inc. Anti-β-actin−peroxidase antibody (AC15) (1:20000), anti-Flag-peroxidase antibody (1:2000) (A8592), anti-HA−peroxidase antibody (H6533) (1:1000), anti-Flag agarose beads (A2220), anti-HA agarose beads (A2095), anti-mouse secondary antibody (A4416) (1:2000), and anti-rabbit secondary antibody (A-4914) (1:2000) were purchased from Sigma-Aldrich. All western blots shown are representatives of three independent experiments. Most important uncropped blot are shown in Supplementary Fig. 5. cAMP levels were measured by the cAMP Direct Immunoassay Kit (ab65355) (Abcam, Cambridge, MA, USA) following Abcam’s protocol, and optical density at 450 nm were measured.

### Immunohistochemistry and tumor analysis

Immunohistochemistry was performed as previously described^9,34,56^. Briefly, 10% formalin solution was used to fix mouse melanomas at 4 °C for overnight. Then the samples were paraffin-embedded and cut into 5-μm-thick sections at Boston University School of Medicine core facility. For antigen retrieval, sections were heated in a boiling water bath for 20 min in 10 mM pH 6.0 sodium citrate buffer. TBS with 0.1% Tween-20 and 5% normal goat serum was used to block the sections. Then tissue sections were incubated with anti-S-100 (1:200) (Dako North America, Inc. Carpinteria, CA, USA) primary antibody at 4 °C overnight and subsequently incubated with secondary antibody and DAB substrate (DAB/Metal Concentrate and Stable Peroxide Substrate Buffer, Thermo Fisher Scientific Inc., Waltham, MA, USA). Lastly, coverslips were mounted by Permount (Thermo Fisher Scientific Inc., Waltham, MA, USA). For mutation measurement, genomic DNA was extracted from paraffin-embedded mouse melanoma sections by using the QIAamp DNA FFPE Tissue Kit (Qiagen, Hilden, Germany). PCR products covering selected mutations were sent directly for DNA sequencing as described^36^.

### Pigment measurement

The whole back skin of mice was collected for eumelanin/pheomelanin measurement. Eumelanin and pheomelanin were quantified by high-performance liquid chromatography based on the level of pyrrole-2,3,5-tricarboxylic acid (PTCA) by alkaline hydrogen peroxide oxidation of eumelanin and 4-amino-3-hydroxyphenylalanine (4-AHP) by hydriodic acid reductive hydrolysis of pheomelanin, respectively. Final results were determined by a conversion as described (eumelanin = 45 × PTCA, pheomelanin = 9 × 4-AHP)^57^. All results are calculated by three independent experiments.

### Clonogenic survival and soft agar assay

The clonogenic survival and soft agar assay were performed as previously described^14,40^. Briefly, engineered hTERT/p53DD/CDK4(R24C)/BRAF^V600E^ melanocytes were exposed to 20 J/m^2^ UVB with pretreatment of 1 μM α-MSH before plating into 6-well plate at 1000 cells per well. Fifteen days after UVB treatment, crystal violet was used to stain colonies. For soft agar assay, 10,000 cells were seeded in 0.5% low-melting-point agarose in Dulbecco’s modified Eagle’s medium (DMEM) with 10% fetal bovine serum (FBS), layered onto 0.8% agarose in DMEM with 10% FBS. After 30 days, the colonies >50 μm were counted under a light microscope. All results are calculated by three independent experiments.

### Bioinformatics analysis

Pearson’s correlation between ZDHHC13 and MITF or DCT in TCGA SKCM and melanoma patient survival analysis based on ZDHHC13 expression (cutoff = 50%) were calculated by GEPIA (http://gepia.cancer-pku.cn/)^24^.

### Statistical analyses

Student’s *t* test was performed for all quantitative data between different groups, and the statistical significance was labeled as *p < 0.05, **p < 0.01, ***p < 0.001. All quantitative data were presented as the mean ± s.d. or ± s.e.m as labeled of at least three independent experiments.

### Reporting summary

Further information on experimental design is available in the Nature Research Reporting Summary linked to this article.

## Supplementary information

Supplementary Information

Reporting Summary

## Data Availability

All data that support the findings of this study are available on reasonable request to the corresponding authors. The contributing authors declare that all relevant data are included in the paper and its supplementary information files.
