# Supplementary material for: Targeting MC1R depalmitoylation to prevent melanomagenesis in redheads
Source: Nat Commun. 2019 Feb 20;10:877. doi: 10.1038/s41467-019-08691-3 (PMC6382811; doi:10.1038/s41467-019-08691-3)
Supplement: Supplementary file 1 — Supplementary Information [file 41467_2019_8691_MOESM1_ESM.pdf]

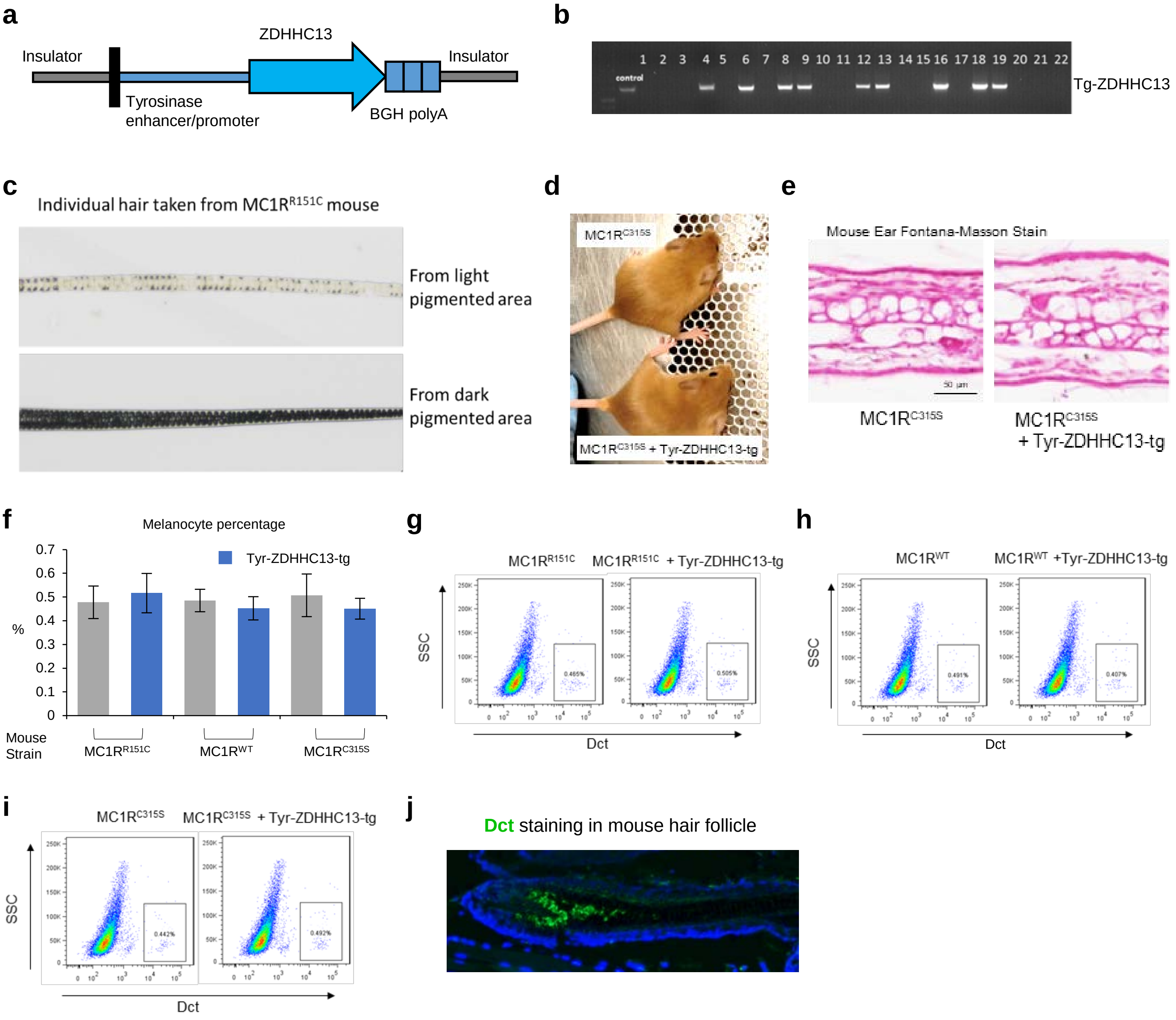

Supplementary Figure 1. The phenotypes of transgenic ZDHHC13 mice.

(a) Schematic diagrams of the ZDHHC13 transgenic construct. **(b)** Genotyping performed to select mice with transgenic ZDHHC13. **(c)** Individual hair taken from MC1R^{R151C} mouse. **(d)** C57/BL6J MC1R variant mice crossed with transgenic ZDHHC13 mice. **(e)** Fontana-Masson staining of indicated ear sections. **(f-i)** Quantification of melanocytes from transgenic mice. Whole back skins from indicated mouse strains were collected and stained with antibody against Dct. Melanocytes were isolated and quantified by FACS. **(j)** Dct immunofluorescence staining in mouse hair follicle. Error bars represent ± s.d.

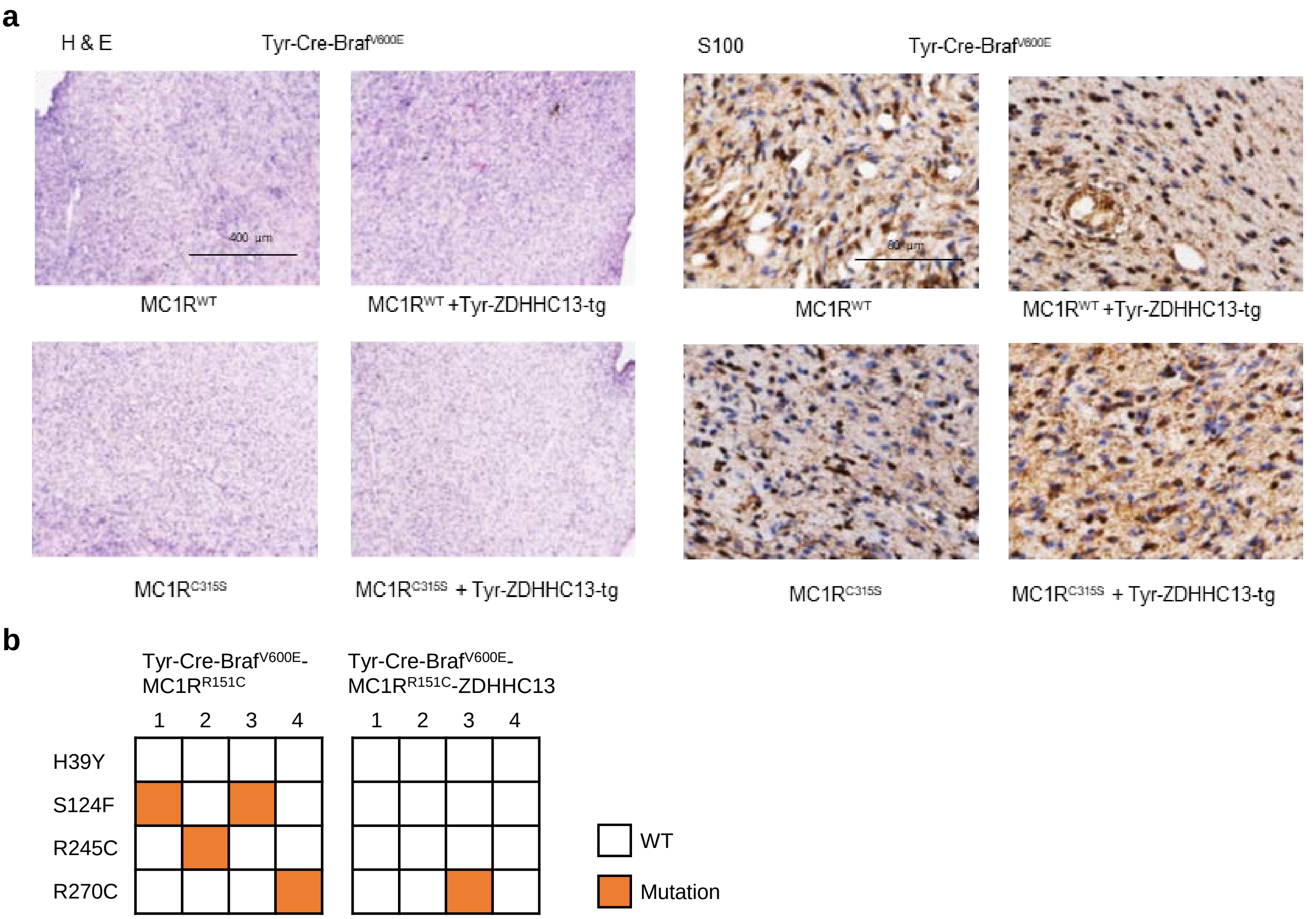

Supplementary Figure 2. UVB-induced melanomagenesis in mouse model.

(a) H&E staining of histological sections and immunohistochemistry staining of S100 of representative cutaneous melanomas. Genotypes were as indicated. **(b)** Trp53 mutations in mouse melanomas in Tyr-Cre-Braf^{V600E}-MC1R^{R151C} and Tyr-Cre-Braf^{V600E}-MC1R^{R151C}-ZDHHC13 mice melanomas.

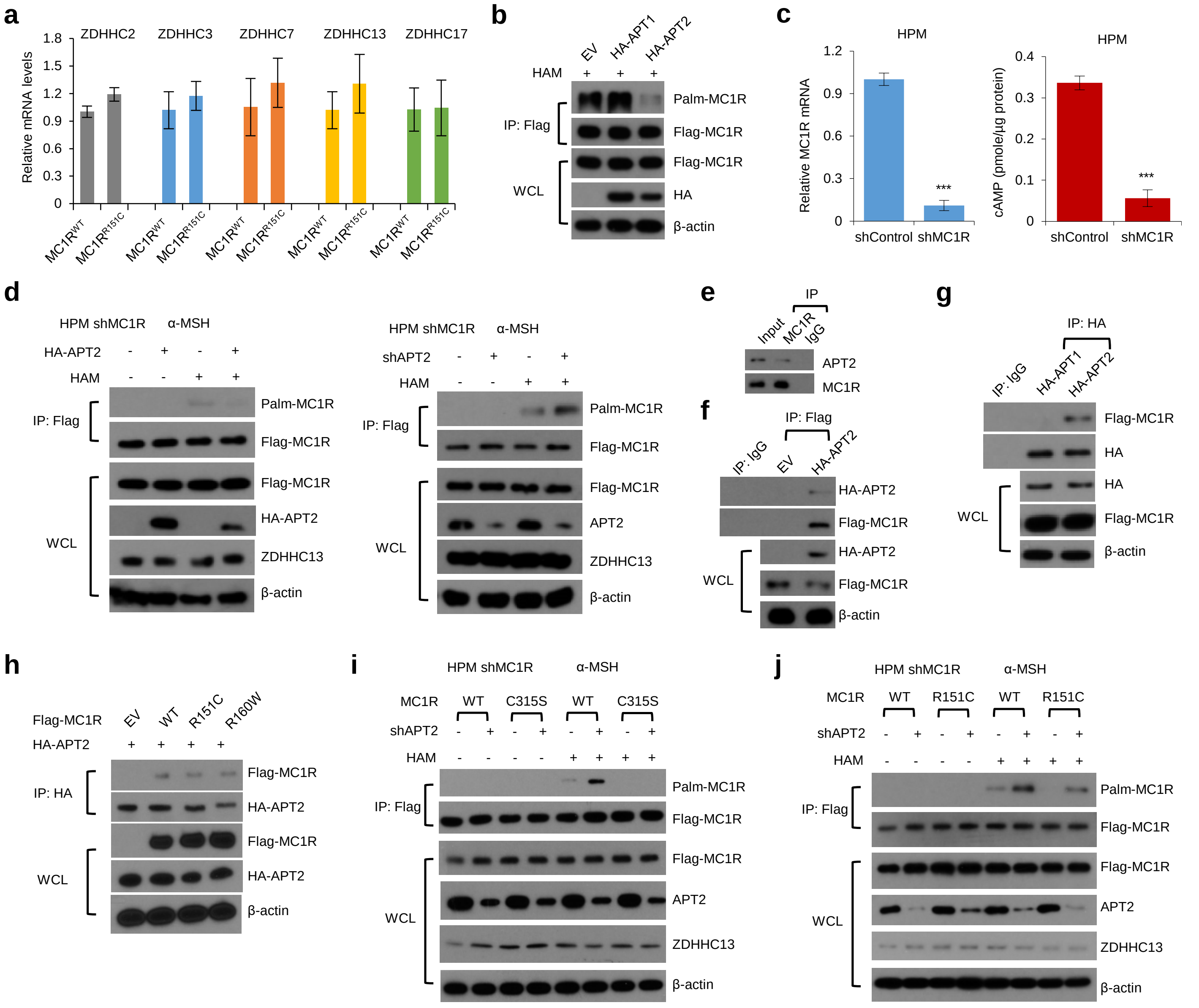

Supplementary Figure 3. APT2 is a depalmitoylating enzyme of MC1R.

(a) The mRNA levels of ZDHHC2, 3, 7, 13, 17 in melanocytes with different MC1R status. **(b)** HPMs were infected with HA-APT1 or HA-APT2 expression vectors and Flag-MC1R encoding retroviral constructs, and cells then were treated with 1 μM α-MSH for 3.5 h. The resulting cells were harvested for IP, ABE and IB analysis with the specific antibodies as indicated. **(c)** HPMs with stable depletion of MC1R by shRNA were treated with 1 μM α-MSH for 3.5 h. The resulting cells were harvested for qRT-PCR and a cAMP immunoassay. **(d)** HPMs with stable depletion of MC1R by shRNA were infected with HA-APT2 or shAPT2 and the Flag-MC1R encoding retroviral constructs, and then cells were treated with 1 μM α-MSH for 3.5 h. The resulting cells were harvested for IP, ABE and IB analysis with the specific antibodies as indicated. **(e)** HPM lysate were immunoprecipitated by anti-MC1R antibodies, and processed for IP and IB analysis using indicated antibodies. **(f)** HPMs were infected with HA-APT2 and Flag-MC1R encoding retroviral constructs. The resulting cells were harvested for IP and IB analysis with specific antibodies as indicated. **(g)** HPMs were infected with HA-APT expression vectors and Flag-MC1R encoding retroviral constructs. The resulting cells were harvested for IP and IB analysis with specific antibodies as indicated. **(h)** HPMs were infected with HA-APT2 expression vectors and Flag-MC1R variants encoding retroviral constructs. The resulting cells were harvested for IP and IB analysis with specific antibodies as indicated. **(i-j)** HPMs with stable depletion of MC1R by shRNA were infected with shAPT2 and retroviruses expressing the indicated Flag-MC1R variants, and were then treated with 1 μM α-MSH for 3.5 h. The resulting cells were harvested for IP, ABE and IB analysis with the specific antibodies as indicated. *$p < 0.05$, **$p < 0.01$, ***$p < 0.001$, unpaired Student's t-test. Error bars represent ± s.d.

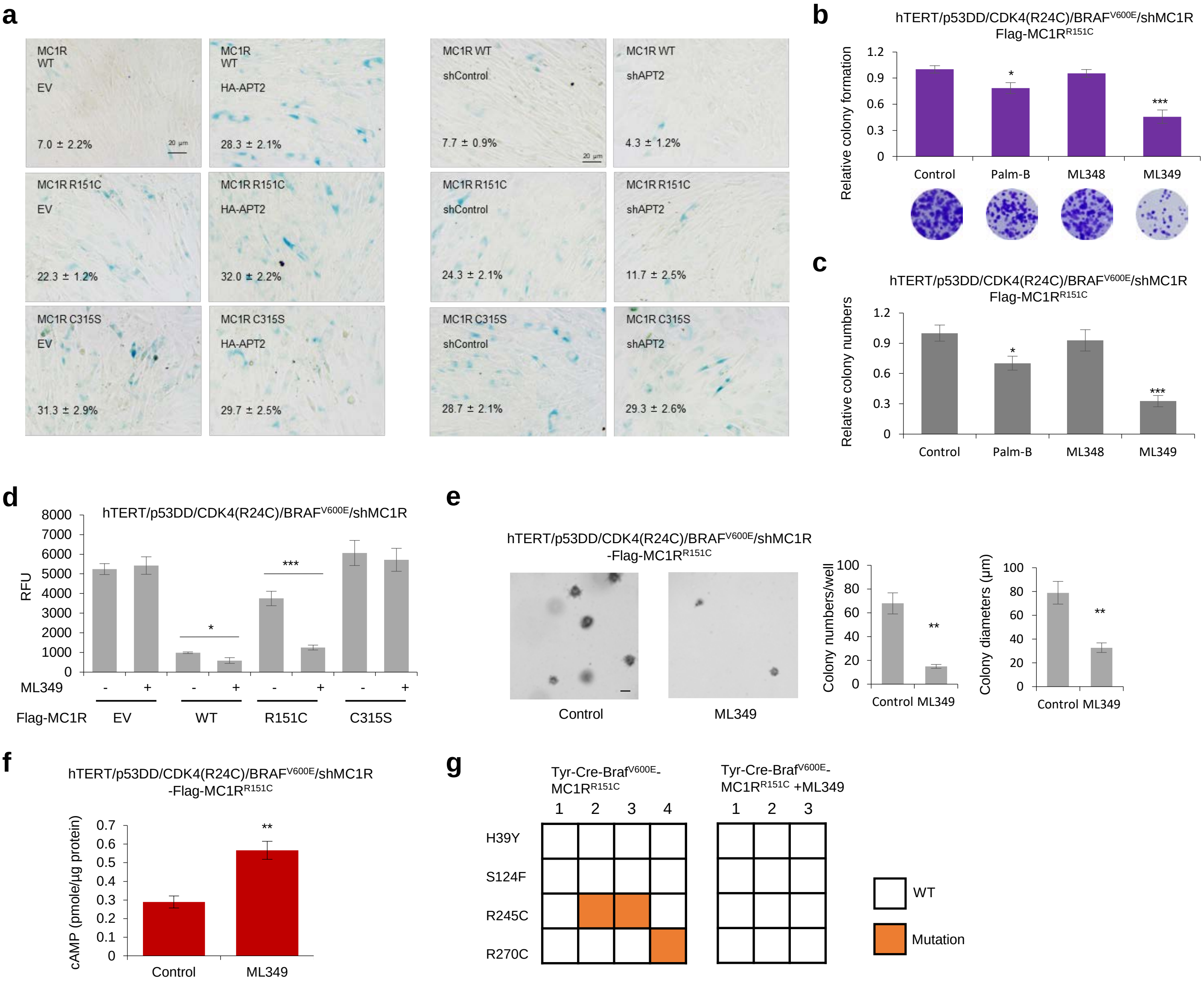

Supplementary Figure 4. APT2 inhibition suppresses UVB-induced melanomagenesis.

(a) HPMs with stable depletion of MC1R by shRNA were infected with viruses expressing HA-APT2 or shAPT2 and retroviruses encoding the indicated Flag-MC1R variants prior to treatment with 1 μM α-MSH for 30 min followed by 25 J/m^2 UVB irradiation. The resulting cells were subjected to SA-β-gal staining assay 7 days after UVR. Data shown correspond to one representative experiment out of three independent experiments. **(b-f)** MC1R-depleted hTERT/p53DD/CDK4(R24C)/BRAF^{V600E} melanocytes were further infected with the indicated Flag-MC1R encoding retroviral constructs. Cells were pre-incubated with 1 μM α-MSH and 100 nM inhibitors for 30min before being irradiated with 20 J/m^2 UVB. **(b)** Cells were subjected to clonogenic survival assays 15 days after UVR. Crystal violet was used to stain colonies. Relative colony numbers were calculated as mean ± SD, n=3. **(c-e)** Cells were seeded (10,000 cells per well) in 0.5% low-melting-point agarose in DMEM with 10% FBS, layered onto 0.8% agarose in DMEM+10% FBS. **(c)** Plates were cultured for 30 days whereupon the colonies >50 μm were counted under a light microscope. Colony numbers were plotted as mean ± SD from three independent experiments. **(d)** Agar was solubilized and the cells were collected for quantification by CyQuant assay. Data are means ± SD, n=3. **(e)** The number of colonies (diameter>50 μm) per well and the average diameters (10 random fields under microscope per well) were counted. **(f)** cAMP levels in indicated cells 3h after UVB irradiation. The resulting cells were harvested for a cAMP immunoassay. The data were compiled from three independent experiments. Data represents three independent experiments. **(g)** Trp53 mutations in mouse melanomas in Tyr-Cre-Braf^{V600E}-MC1R^{R151C} and Tyr-Cre-Braf^{V600E}-MC1R^{R151C} + ML349 mice melanomas. *$p < 0.05$, **$p < 0.01$, ***$p < 0.001$, unpaired Student's t-test. Error bars represent ± s.d.

Fig. 2g

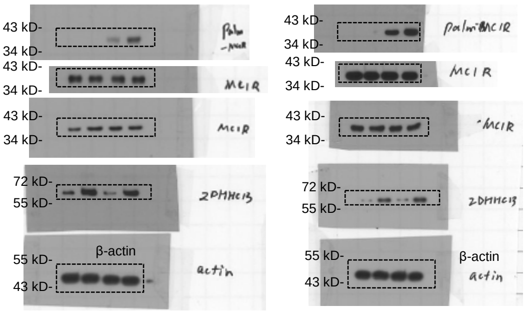

Fig. 4c

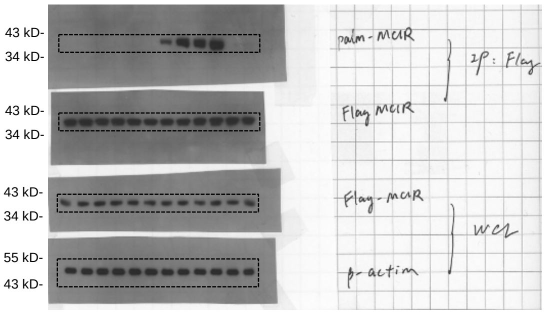

Supplementary Figure 5. Unprocessed scans of the most important blots
